# Sondenlose Herzschrittmacher: Eine „dauerhaft“ gute Lösung?

**DOI:** 10.1007/s00399-023-00970-3

**Published:** 2023-10-23

**Authors:** Clemens Steinwender, Hermann Blessberger, Karim Saleh

**Affiliations:** 1 Klinik für Kardiologie und Internistische Intensivmedizin, Kepler Universitätsklinikum Linz, Medizinische Fakultät, Johannes Kepler Universität Linz, Linz, Österreich; 2 Klinik für Kardiologie und Internistische Intensivmedizin, Klinisches Forschungsinstitut für Kardiovaskuläre und Metabolische Erkrankungen, Kepler Universitätsklinikum Linz, Medizinische Fakultät, Johannes Kepler Universität, Krankenhausstr. 9, 4021 Linz, Österreich

**Keywords:** Schrittmacherimplantation, Komplikationen, Risikoreduktion, Infektion, Langzeitergebnisse, Pacemaker Implantation, Complications, Risk reduction, Infection, Long-term outcome

## Abstract

Die beiden aktuell erhältlichen sondenlosen Schrittmacher weisen in den jeweiligen Zulassungsstudien sowie den zusätzlichen Real-world-Registern eine hohe Effektivität und Sicherheit auf. Im Vergleich zu konventionellen Schrittmachern finden sich niedrigere Langzeit-Komplikationsraten (v. a. im Hinblick auf Sondendislokationen und Systeminfektionen). Zunehmende Evidenz (derzeit größtenteils für den Micra^TM^ [Medtronic, Minneapolis, MN, USA]) zeigt, dass diese Vorzüge auch Bestand haben und daher von einer „dauerhaft guten Lösung“ gesprochen werden kann.

## Abstract (en)

The two currently available leadless pacemakers are highly effective and safe in the respective approval studies and also real-world registries. Compared to conventional pacemakers, there are lower long-term complication rates compared to conventional pacemaker systems (especially regarding lead dislocations and systemic infections). Increasing evidence (currently largely for the Micra™ [Medtronic, Minneapolis, MN, USA] device) shows that these advantages are also valid in the long-term. Leadless pacemakers can therefore be regarded a “permanently good solution”, when appropriately implanted in suitable patients.

Seit der ersten Implantation eines Herzschrittmachers in den 1950er-Jahren hat die Schrittmachertechnologie zahlreiche Entwicklungen (z. B. verbesserte Batteriekapazität, subtilere Stimulationsalgorithmen, programmierbare Sensoren zur Steuerung der Stimulationsfrequenz, MRT-Tauglichkeit) durchlaufen [1]. Das technologische Konzept der Generierung und Abgabe des Schrittmacherstimulus mittels infraklavikulär implantiertem Impulsgenerator sowie von dort via Venen zum Herz reichenden Sonden blieb lange unverändert, obwohl diese die Schwachstellen der konventionellen Schrittmachertherapie sind. Entsprechende Komplikationen (Hämatome/Infektionen im Bereich des Generators, Dislokationen, Brüche und Infektionen der Sonden) treten bei 7–12 % aller PatientInnen auf und führen in ca. 4 % zu chirurgischen Eingriffen [2, 3].

Sondenlose Schrittmacher wurden entwickelt, um dieses Risiko zu reduzieren. Dabei sind sämtliche Funktionseinheiten des Schrittmachers in einer kleinen Kapsel untergebracht. Durch ihre geringe Größe ist eine kathetergestützte Implantation möglich, ohne das zuführende venöse System oder die Trikuspidalklappe mit Sonden dauerhaft belasten zu müssen.

Derzeit sind in der EU folgende sondenlose Schrittmacher kommerziell erhältlich: Micra^TM^ (Medtronic Inc., Minneapolis, MN, USA) mit entweder VVI(R)-Stimulationseigenschaften (Micra^TM^ VR) oder VDD(R)-Stimulationseigenschaften (Micra^TM^ AV) sowie Aveir^TM^ VR (Abbott, Plymouth, MN, USA) mit VVI(R)-Stimulationseigenschaften. Diese Geräte sind auch in allen Ländern, die einen FDA-Approval akzeptieren, für die klinische Verwendung zugelassen. Zusätzlich ist in den USA auch bereits das DDD(R)-System AVEIR^TM^ DR (Abbott, USA) mit je einem sondenlosen Schrittmacher im rechten Vorhof und rechten Ventrikel (drahtlose Implantat-zu-Implantat-Kommunikation) zur Implantation freigegeben. Eine im Mai 2023 im *New England Journal of Medicine* publizierte Studie hat bei 300 PatientInnen ein gutes Sicherheitsprofil und eine hohe Effektivität dieser Technologie ergeben und zur US-Zulassung, die in der EU noch zumindest ein Jahr dauern dürfte, geführt [4].

Die ersten Micra^TM^ VR wurden im Rahmen der Zulassungsstudien im letzten Quartal 2013 implantiert, weshalb nun Ende 2023 deren 10-jährige Laufzeit erstmals überschritten wird. Es ist daher gerechtfertigt, die Frage zu beantworten, ob die Implantation sondenloser Schrittmacher bei geeigneten PatientInnen eine „dauerhaft“ gute Lösung darstellt.

## Technischer Aufbau und Laufzeit

Micra^TM^ VR und AV beinhalten in einer baugleichen zylindrischen Hülle aus Titan alle Bauteile eines 1,5 und 3 T full-body-MR-kompatiblen VVI(R)-Schrittmachers. Die Abmessungen von 26 × 7 mm ergeben ein Volumen von 0,8 cm^3^ und ein Gewicht von 2 g. Der Micra^TM^ AV benutzt die zur Frequenzmodulation integrierten Akzelerometer, um bei PatientInnen im Sinusrhythmus (SR) die Kontraktion der Vorhöfe zu detektieren und im Fall einer AV-Überleitungsstörung ein getriggertes Ventrikelpacing zu ermöglichen. Diese Funktion wird durch den sog. MARVEL-Algorithmus vermittelt, der in der MARVEL-2-Studie erfolgreich getestet wurde [5].

Die Batterie von Micra^TM^ VR und AV hat eine Kapazität von 120 mAH und eine prognostizierte Laufzeit von 12–13 Jahren, unterstützt durch eine automatische Reizschwellenbestimmung mit entsprechender Stimulations-Amplitudenanpassung.

Die 4 elektrisch inaktiven Verankerungshaken (Tines) aus Nitinol sind am distalen Ende rund um die Elektrode gruppiert. Deren technischer Aufbau entspricht dem einer konventionellen steroidabgebenden passiven Stimulationskathode mit einer Oberfläche von 2,5 mm^2^.

**Figure 1:**
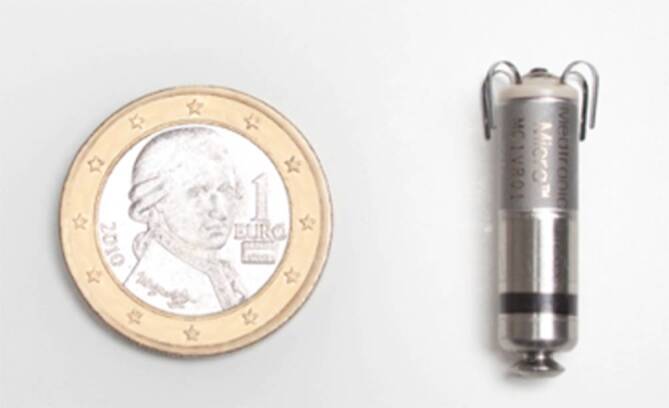

Das proximale Ende des Gehäuses ist pilzförmig gestaltet, um im Fall einer notwendigen Bergung das Fassen mit Snares zu erleichtern (Abb. 1).

Der Micra^TM^ ist konventionell über den Medtronic-Programmer abfrag- und programmierbar. Auch eine telemedizinische Überwachung mittels CareLink^TM^ ist möglich.

**Figure 2:**
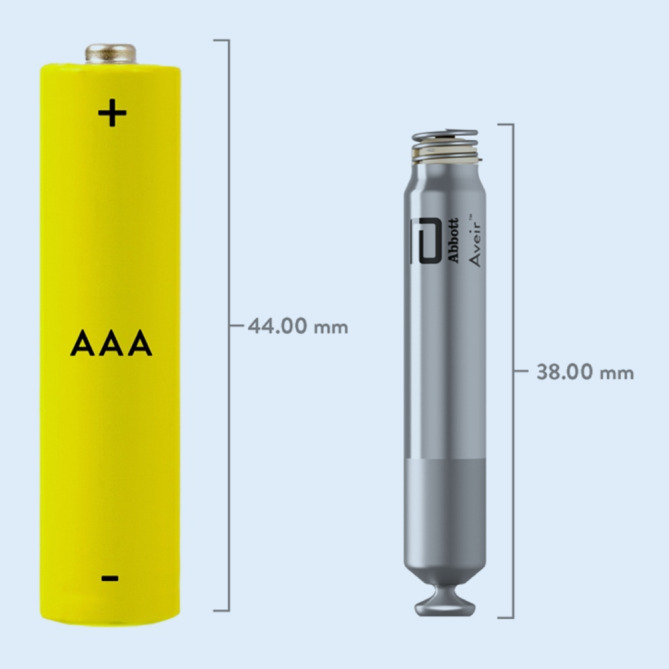

Der Aveir^TM^ VR ist ebenfalls ein sondenloser VVI(R)-Schrittmacher, der 1,5 und 3 T full-body-MR-kompatibel ist. Die Abmessungen der zylindrischen Titanhülle sind 38 × 6,5 mm, das Gewicht beträgt 2,4 g, das Volumen 1,1 cm^3^. Mittels eines Thermo-Sensors basiert die Frequenzsteuerung auf der Messung geringfügiger Temperaturanstiege des Blutes bei Belastungsbeginn. Die Batteriekapazität des AVEIR^TM^ VR beträgt 243 mAH mit einer prognostizierten Laufzeit von 17–18 Jahren. Das Gerät verfügt über keine automatische Reizschwellenbestimmung. Die Fixierung des Schrittmachers erfolgt über eine elektrisch inaktive Helix, die im Zuge einer 1,5fachen Umdrehung etwa 1,6 mm in das Endo‑/Myokard eingeschraubt wird. Im Zentrum der Helix sitzt die steroidabgebende passive Stimulationskathode mit einer Oberfläche von 2 mm^2^. Ebenso wie beim Micra^TM^ befindet sich am proximalen Ende des Gehäuses eine Struktur für das interventionelle Fassen des Geräts (Abb. 2).

Der Aveir^TM^ ist konventionell über den Abbott-Programmer abfrag- und programmierbar. Dafür ist ebenso wie bei der Implantation die Verwendung des sog. Aveir^TM^ Link Module erforderlich, welches eine sehr stromsparende konduktive Kommunikation ermöglicht. Eine telemedizinische Überwachung ist derzeit nicht möglich. Von besonderem Interesse ist, dass die bereits derzeit implantierten Geräte auf ein sondenloses Zweikammer-Schrittmacher-System (Aveir^TM^ DR) aufgerüstet werden können.

## Implantation

**Figure 3:**
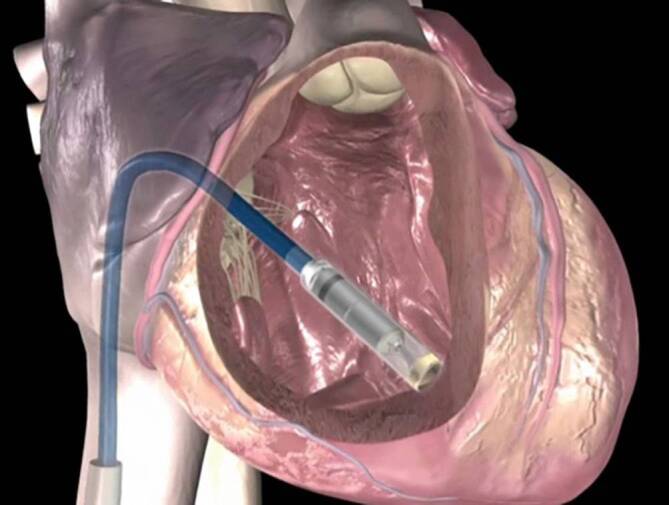

Der Micra^TM^ und der Aveir^TM^ werden jeweils mit einem Delivery-System geliefert. Dies ist ein Katheter, an dessen Spitze der Schrittmacher in einem Zylinder untergebracht ist (der Micra^TM^ ist vormontiert, während der Aveir^TM^ am Interventionstisch „geladen“ wird). Das Delivery-System wird in einer Schleuse (23 F Innen- und 27 F Außendurchmesser) über die V. femoralis, V. iliaca externa und V. cava inferior in das rechte Atrium vorgeschoben. Mittels eines unidirektionalen Krümmungsmechanismus des Delivery-Systems kann dieses durch die Trikuspidalklappe in den rechten Ventrikel manövriert werden (Abb. 3). Gemäß den Empfehlungen der Hersteller und publizierter Evidenz werden Micra^TM^ VR und AV eher mittseptal, der Aveir^TM^ inferoseptal implantiert.

Wird ein Kontakt des Zylinders am Myokard erzielt (durch Kontrastmittel-Injektion über das Delivery-System visualisierbar), kann die Implantation des Schrittmachers erfolgen. Bei beiden Systemen wird dafür der Zylinder zurückgezogen. Beim Micra^TM^ werden dadurch die Tines freigesetzt, die sich im Endo‑/Myokard verhaken. Beim Aveir^TM^ beginnt hingegen erst nach dem Zylinderrückzug der eigentliche Fixierungsvorgang durch ein kathetervermitteltes Einschrauben ins Endo‑/Myokard. Durch diesen Zwischenschritt kann beim Aveir^TM^ vor der eigentlichen Fixation ein Mapping erfolgen, bei dem die elektrischen Parameter (Sensing, Reizschwelle, Impedanz) der Implantationsstelle evaluiert werden.

Nach der Fixierung (die Schrittmacher sind noch über *Tethers* mit dem Delivery-System verbunden) erfolgen manuelle Manöver zum Nachweis einer guten mechanischen Verankerung und die definitive Bestimmung der elektrischen Parameter. Ist eine Repositionierung erforderlich, wird der Schrittmacher mithilfe der Tethers wieder ans Delivery-System angedockt und eine neue Implantationsstelle angesteuert. Sind die Parameter zufriedenstellend, werden die Tethers gekappt und der Schrittmacher damit vollständig abgesetzt. Danach werden Delivery-System und Schleuse aus dem Körper gezogen und die Punktionsstelle meist mit einer besonderen Hautnaht (Z- oder Tabaksbeutel-Naht) versorgt.

## Klinische Daten

### Micra^TM^ VR.

Die prospektive nichtrandomisierte multizentrische Micra DIE-Studie untersuchte den Micra^TM^ VR bei 726 PatientInnen [6]. Die Implantation verlief dabei bei 99,2 % (719/726) erfolgreich. Komplikationen traten bei 3,4 % auf: Perikarderguss oder -tamponade bei 1,5 %, vaskuläre Komplikationen bei 0,7 % und erhöhte Stimulationsreizschwellen im 6‑Monats-Follow-up bei 0,3 %. Der einzige Todesfall war durch eine Niereninsuffizienz verursacht, also nicht gerätebezogen. Duray et al. konnten für dieses PatientInnen-Kollektiv zeigen, dass die Komplikationsrate bei einem längeren Follow-up von 12 bis 18 Monaten lediglich auf insgesamt 4 % stieg. Diese war dabei im retrospektiven Vergleich zu einem Kollektiv von 2667 Patienten mit konventionellen Schrittmachern um 48 % erniedrigt, was v. a. auf das völlige Fehlen von Dislokationen und Infektionen des Micra^TM^ VR zurückgeführt werden konnte. Die niedrige Komplikationsrate resultierte in einer um 47 % reduzierten Hospitalisierungsrate [7]. Die Ergebnisse dieser Studie wurden in Real-world-Registern von Roberts et al. bzw. El-Chami et al. bestätigt, wobei von Letzteren mittels öffentlicher US-Gesundheitsakten 6219 PatientInnen mit Micra^TM^ VR und 10.212 PatientInnen mit konventionellen VVIR-Schrittmachern in einem 2‑Jahres-Follow-up-Zeitraum miteinander verglichen wurden. Auch hier zeigte sich bei vergleichbarer Mortalität eine geringere Langzeit-Komplikationsrate für sondenlose Schrittmacher (31 % niedrigere chronische Komplikationsrate im Vergleich zu konventionellen Systemen; [8, 9]). Insgesamt ist für den Micra^TM^ VR im Vergleich zu konventionellen Schrittmachersystemen von einer gering höheren Rate an Myokardverletzungen bei der Implantation, jedoch einer niedrigeren Rate an mittel- und langfristigen Komplikationen – insbesondere im Bereich der Generatorloge und der Sonden – auszugehen [7–9]. Zu beachten ist jedoch, dass die vorhandenen Langzeitdaten auf den Micra^TM^ VR limitiert sind und die Follow-up-Dauer meist nur 2 Jahre beträgt. Des Weiteren soll hier noch einmal betont werden, dass bislang keine randomisierten Studien existieren, in denen konventionelle und sondenlose Schrittmacher verglichen werden.

### Micra^TM^AV.

Da der Micra^TM^AV bis auf den in die Elektronik integrierten MARVEL-Algorithmus dem Micra^TM^ VR baulich gleicht, sind die für den VR beschriebenen klinischen Ergebnisse auf den AV sicherlich übertragbar.

Die Akzelerometer-mediierte mechanische Wahrnehmung der Vorhofkontraktion des VDD-Algorithmus weist jedoch im Vergleich zu konventionellen Systemen mit elektrischer Wahrnehmung der Vorhoferregung v. a. bei höheren Herzfrequenzen geringere Raten an atrioventrikulärer (AV) Synchronität auf. Dies macht oft repetitive Optimierungen der Programmierung erforderlich, wobei die getrackte Sinusfrequenz meist unter 115/min bleibt, bevor das System in einen VVIR-Modus umschaltet [10, 11].

Aktuell ist weltweit eine deutliche Zunahme von Conduction-System-Pacing (CSP) zur Verhinderung einer Pacing-induzierten Kardiomyopathie zu verzeichnen. Da CSP mit den aktuell verfügbaren sondenlosen Schrittmachern nicht möglich ist, hat die Inzidenz einer Pacing-induzierten Kardiomyopathie bei PatientInnen mit sondenlosen Schrittmachern besondere Bedeutung. Mehrere rezent publizierte Arbeiten haben sich dieser Frage angenommen und berichten über (auch im Vergleich zu konventionellen VVI-Schrittmachern) niedrige Raten an Pacing-induzierten Kardiomyopathien von 3,0–7,8 %, v. a. bei einer mitt- bis hochseptalen Position der sondenlosen Schrittmacher. Dementsprechend fand sich auch eine sehr niedrige Rate an neu implantierten Systemen zur kardialen Resynchronisationstherapie (CRT-Systeme, in die sondenlose Schrittmacher derzeit nicht integriert werden können, also stillgelegt werden müssen; [9, 12, 13]).

### Aveir^TM^ VR.

Der technologische Vorläufer des AveirTM VR, der sondenlose VVI(R)-Schrittmacher NanostimTM (St. Jude Medical, USA), wurde in der LEADLESS- bzw. LEADLESS-II-Studie erfolgreich getestet und war daraufhin der erste kommerziell erhältliche sondenlose Schrittmacher, bevor er insbesondere wegen Batterieproblemen vom Markt genommen werden musste [14, 15]. Der Aveir^TM^ VR selbst wurde nach substanziellen Modifikationen des Nanostim^TM^, v. a. an Batterie und „docking button“, im LEADLESS II (Phase 2) Trial getestet. Die Implantation verlief dabei bei 98 % (205/210) der PatientInnen erfolgreich. Komplikationen traten in einem 1‑Jahres-Follow-up bei 7 % (195/210) auf, davon periinterventionelle Perikardergüsse/-tamponaden bei 1,9 % (4/210). Todesfälle waren nicht zu beklagen. Die elektrischen Parameter zeigten sich auch nach einem Jahr stabil. Die positiven Ergebnisse führten schlussendlich zu einem FDA-Approval und einer Zulassung in Europa (CE mark; [16, 17]).

## Laufzeit

### Micra^TM^.

Die nominale Laufzeit beträgt 12 bis 13 Jahre beim VR, 1 bis 1,5 Jahre kürzer beim AV. Breeman et al. konnten in einer Analyse von 153 PatientInnen mit einem Micra^TM^ VR bei einem mittleren Follow-up von 35 Monaten (bis maximal 7 Jahre) nachweisen, dass bei einem Großteil der PatientInnen sogar mit einer Laufzeit von > 13 Jahren (bei einem medianen Stimulationsanteil von 35 % und einer stabilen mittleren Stimulationsreizschwelle von 0,7 V @0,24 ms) gerechnet werden kann. Batterieversagen im Sinne eines unvorhergesehenen signifikanten Abfalls der Batteriekapazität traten nicht auf [18]. Außerhalb Europas ist bereits derzeit eine Nachfolge-Generation des Micra^TM^ verfügbar, Micra^TM^ VR2 und AV2, die beide nun durch eine größere Batteriekapazität projektierte mediane Laufzeiten von 16,7 Jahren (VR) bzw. 15,6 Jahren (AV) aufweisen.

### Aveir^TM^.

Die nominale Laufzeit beträgt 17 bis 18 Jahre. Im Juli 2023 wurden von Reddy et al. die Ergebnisse der 1‑Jahres-Follow-ups des LEADLESS II (Phase 2) Trials publiziert, bei denen eine erwartete mittlere Laufzeit von 17,6 ± 6,6. Jahren ermittelt wurde. Diese Analyse schätzt den Anteil der Geräte mit einer Laufzeit von > 10 Jahren auf 83 % und von > 20 Jahren auf 48 % [17]. Micra^TM^ und Aveir^TM^ können zum Ende der Batterielaufzeit per Programmierung komplett deaktiviert werden. Damit ist prinzipiell die Implantation eines zweiten sondenlosen Schrittmachers möglich, der dann ohne funktionelle Interferenz aktiviert werden kann. Die anatomischen Voraussetzungen für die Implantation eines zweiten Micra^TM^ ohne physische Interaktion zwischen beiden Geräten scheinen im rechten Ventrikel erfüllt zu sein [19].

## Extraktion

Für die Notwendigkeit einer Bergung haben Micra^TM^ und Aveir^TM^ an ihrem proximalen Ende ein sog. *Retrieval Feature *bzw. einen *Retrieval Button*. Hier können sie interventionell mit Snares gefasst und extrahiert werden. Für den Aveir^TM^ ist ein spezieller Retrieval-Katheter verfügbar, für den Micra^TM^ nicht. Der für den Aveir^TM^ erhältliche Retrieval-Katheter wurde wegen der Batterieprobleme des baulich ähnlichen Vorläufermodells Nanostim^TM^ bereits häufig eingesetzt. So zeigte sich bei 241 PatientInnen eine generelle Extraktionserfolgsrate bei chronisch (3,1 ± 1,8 Jahre) implantierten Geräten von 88 % und dabei ein niedriges Risiko für Perikard-Effusionen/-Tamponaden von 0,8 % [20].

Etwas komplexer stellt sich die Situation beim Micra^TM^ dar. Eine Micra^TM^-Bergung mit dem Delivery-System ist nur während einer laufenden Implantationsprozedur oder nach Implantation eines zweiten Micra^TM^ vor Bergung des ersten möglich. Dann kann ein Snare durch den Tether-Kanal des Delivery-Systems vorgebracht, der Micra^TM^ damit gefasst und in den Cup des Delivery-Systems zurückgezogen werden. Ohne Delivery-System wird ein Micra^TM^ über die originale Schleuse (diese ist erhältlich) und eine darin vorgebrachte steuerbare Schleuse geborgen. Zu beachten ist, dass dabei der Micra^TM^ ohne schützendes Delivery-System aus dem Myokard extrahiert und durch die Trikuspidalklappe zur im Vorhof liegenden Schleuse gezogen wird. Machbarkeit und Sicherheit dieser Manöver konnten u. a. in einer Übersicht über 40 Fälle von Micra^TM^-Extraktionen belegt werden [21, 22].

Dislokationen von sondenlosen Schrittmachern mit frei flottierenden Geräten im Ventrikel oder einer Embolisation derselben wurden in den Zulassungsstudien nicht beobachtet und auch danach nur als Raritäten publiziert [23, 24]. Bei korrekter Implantation gilt der Verankerungsmechanismus beider Modelle daher auch langfristig als sicher.

## Implantation bei Patienten mit hohem Infektionsrisiko oder nach Infektion

Sowohl in der IDE-Studie als auch im Micra Post Approval Registry traten keine Infektionen des Micra^TM^ auf [6, 8]. Als Grund dafür kommen die geringe Größe sowie die rasche und meist vollständige Endothelialisierung des Micra^TM^ in Frage [25, 26]. Im Vergleich zur großen Fremdkörperoberfläche konventioneller Herzschrittmachersysteme bietet die Titan‑/Parylenoberfläche des Micra^TM^ wenig Angriffsfläche für bakteriellen Bewuchs. Damit bietet sich das System für PatientInnen mit erhöhtem Infektionsrisiko (Diabetes mellitus, Niereninsuffizienz, chronische Hämodialyse, Immunsuppression etc.) oder bei Zustand nach Extraktion eines konventionellen Systems wegen Infektion an. So konnte gezeigt werden, dass die Implantation des Micra^TM^ nach Systeminfektionen machbar und im Follow-up sicher ist [27, 28].

Für den Aveir^TM^ liegen Daten in dieser Größenordnung noch nicht vor, aufgrund der vergleichbaren Größe, derselben Oberfläche sowie der Tatsache, dass in der publizierten Evidenz über keine infizierten Systeme berichtet wurde, kann jedoch mit großer Wahrscheinlichkeit von einem *Klasseneffekt* gesprochen werden [14–17].

## Indikationen

Die rezenten (2021) ESC-Guidelines sprechen für sondenlose Schrittmacher eine Klasse-IIa-Empfehlung aus, wenn kein konventioneller Zugang (z. B. durch verschlossene Armvenen) möglich oder das Risiko für eine Sondeninfektion hoch ist (z. B. vorhergegangene Infektion oder chronische Hämodialyse). Eine IIb-Empfehlung besteht für eine permanente Einkammer-Stimulation als Alternative zu konventionellen Systemen (in Abhängigkeit von Lebenserwartung, der PatientInnen etc.; [29]).

Am häufigsten wurden sondenlose Schrittmacher im Rahmen der Studien und Register bei älteren PatientInnen mit langsam/blockiert übergeleiteten atrialen Tachykardien sowie erhaltener Linksventrikelfunktion implantiert.

Dies macht aus folgenden Gründen Sinn:

1. Ältere Patienten können wegen ihrer häufigeren Risikofaktoren für Langzeitkomplikationen konventioneller Schrittmachersysteme, wie Diabetes mellitus oder Niereninsuffizienz [2, 3], besonders von sondenlosen Schrittmachern profitieren.
2. Es besteht eine klassische VVIR-Indikation.
3. Sondenlose Schrittmacher können nicht auf ein CRT-System (kardiale Resynchronisationstherapie) aufgerüstet werden. Mit den derzeitigen Systemen kann auch kein CSP erzielt werden.

Durch den Micra^TM^ AV werden auch zunehmend (eher ältere und weniger aktive) PatientInnen im Sinusrhythmus mit höhergradigen AV-Blockierungen implantiert.

Durch die Möglichkeit, bereits jetzt implantierte Aveir^TM^-VR-Geräte in Zukunft durch zusätzliche Implantation eines weiteren Aveir^TM^ im Vorhof auf ein Aveir^TM^-DR-System aufzurüsten, wird sich die Indikation (z. B. auf PatientInnen im SR mit vorerst geplanter *Back-up-Stimulation*) sicher erweitern.

## Fazit für die Praxis

- Mit dem Micra^TM^ und Aveir^TM^ existieren zwei sondenlose Schrittmachermodelle, die in den jeweiligen Zulassungsstudien sowie den zusätzlichen Real-world-Registern eine hohe Effektivität und Sicherheit aufweisen.
- Im Vergleich zu konventionellen Schrittmachern konnten dabei niedrigere Langzeit-Komplikationsraten (v. a. im Hinblick auf Sondendislokationen und Systeminfektionen) gezeigt werden.
- Zunehmende Evidenz (derzeit größtenteils für den Micra^TM^ vorliegend) zeigt, dass diese Vorzüge auch Bestand haben und daher von einer „dauerhaft guten Lösung“ gesprochen werden kann.
